# What can go wrong in the non-coding genome and how to interpret whole genome sequencing data

**DOI:** 10.1515/medgen-2021-2071

**Published:** 2021-08-14

**Authors:** Heiko Krude, Stefan Mundlos, Nancy Christine Øien, Robert Opitz, Markus Schuelke

**Affiliations:** Institute of Experimental Pediatric Endocrinology, Charité–Universitätsmedizin Berlin, Corporate Member of Freie Universität Berlin and Humboldt-Universität zu Berlin, Berlin, Germany; Institute for Medical and Human Genetics, Charité–Universitätsmedizin Berlin, Corporate Member of Freie Universität Berlin and Humboldt-Universität zu Berlin, Berlin, Germany; Department of Neuropediatrics, Charité–Universitätsmedizin Berlin, Corporate Member of Freie Universität Berlin and Humboldt-Universität zu Berlin, Berlin, Germany; NeuroCure Cluster of Excellence, Charité–Universitätsmedizin Berlin, Corporate Member of Freie Universität Berlin and Humboldt-Universität zu Berlin, Berlin, Germany

**Keywords:** genetic disease, non-coding genome, splice site mutations, enhancer mutations, structural variants, non-coding variant evaluation

## Abstract

Whole exome sequencing discovers causative mutations in less than 50 % of rare disease patients, suggesting the presence of additional mutations in the non-coding genome. So far, non-coding mutations have been identified in less than 0.2 % of individuals with genetic diseases listed in the ClinVar database and exhibit highly diverse molecular mechanisms. In contrast to our capability to sequence the whole genome, our ability to discover and functionally confirm such non-coding mutations is lagging behind severely. We discuss the problems and present examples of confirmed mutations in deep intronic sequences, non-coding triplet repeats, enhancers, and larger structural variants and highlight their proposed disease mechanisms. Finally, we discuss the type of data that would be required to establish non-coding mutation detection in routine diagnostics.

## Introduction

The advent of whole exome sequencing (WES) has led to a wave of discovery of underlying genetic causes of human disease. WES covers approximately 2 % of the genome and can be used to detect single-base pair variants and small insertions/deletions (indels) in coding exons and their flanking intronic regions. This approach has accelerated the discovery of genetic disease-causing variants, significantly improved our ability to identify dominant *de novo* mutations [1], and broadened our understanding of the spectrum of different genetic syndromes. Due to the declining cost of large-scale sequencing, WES is at the cusp of becoming part of routine care for patients with suspected genetic disease [2] and negotiations are ongoing with the major health insurance providers to cover the costs. As the first German health insurance contracts for full cost coverage of WES were signed recently, it is clear that WES is becoming part of clinical care in Germany.

The currently available public [3], [4] and commercial data analysis pipelines process WES data quickly and predict variant pathogenicity with an accuracy of 80–90 % [3], [4]. The advent of WES has improved the diagnostic yield in the hands of experienced clinicians and geneticists from 5–25 % for Sanger sequencing of candidate genes to 16–52 % [5], [6]. The current average WES diagnostic rate is 30 %, with a substantial variation of 7–44 % between disease groups [7], [8]. The diagnostic rate for patients with rare genetic disorders is significantly improved by using the Human Phenotype Ontology (HPO) [9] to interpret a patient’s genotype in the context of the clinical phenotype [10]. Here we use the EU definition of “rare genetic disorders” as those disorders occurring with a prevalence of less than 1:2,000.

In spite of the significant improvements in molecular diagnostic rates for rare genetic diseases resulting from the implementation of WES, the molecular diagnostic rates have leveled off at approximately 30–40 % [6], [7]. The reasons for this are many, but are likely to include mutations in the 98 % of the genome not covered by WES such as **(i)** disease-causing variants in regulatory regions, including transcription factor binding sites (TFBS) in enhancers or promoters. Alterations can also affect higher-level chromatin organization through histone modification and DNA methylation or the disruption of chromatin domains (topologically associating domains [TADs]), resulting in a perturbation of promoter/enhancer interactions [11]. **(ii)** WES does not detect pathogenic variants located deep inside introns, outside of splice site flanking sequences. Such variants could result in gain of cryptic splice sites [12]. **(iii)** Standard WES approaches detect indels smaller than 100 bp, while array-CGH detects copy number variations (CNVs) larger than 100 kb depending on resolution. Consequently, our current approaches do not reliably detect small CNVs in the 100 bp to 100 kb range, as well as balanced and more complex genomic rearrangements. The current WES datasets are often of inconsistent quality due to bias introduced by the intermittent capture and enrichment steps integral to WES. Generation of uniform and quantifiable WES results requires highly reproducible handling and processing and the use of identical WES capture kits. Though current platform-specific algorithms aim to interpret WES data with respect to CNVs [13], these factors often make it impossible to use these algorithms for cross-platform comparisons [14]. **(iv)** Due to the short 100–250 bp length of Illumina sequencing reads, longer trinucleotide repeat tracts cannot be satisfactorily resolved, often precluding correct diagnosis of trinucleotide repeat expansion disorders [15]. **(v)** Our understanding of how genetic variants in coding regions of transcription factors (TFs) affect steady-state mRNA and protein levels is limited. This means we are unable to determine whether or not such variants are pathogenic and/or contributing factors to a disease phenotype [16]. Such shortcomings could be overcome by parallel sequence analysis of the whole genome together with RNA sequencing of affected tissues [17], a method for which easy-to-use software is lacking. **(vi)** Variants located in modifying genes may have a compounding effect on the phenotype. This may be particularly significant for variants in those genes involved in multiple signaling or metabolic pathways, with β-catenin as a prominent example [18]. Such additive effects are difficult to pinpoint in the small cohorts typical of rare diseases. **(vii)** We are just starting to understand how structural variants (SVs), such as deletions, inversions, and duplications, exert long range effects on chromatin 3D folding and subsequent gene transcription [19]. Such higher-order rearrangements are currently difficult to predict and improving this ability requires a substantial amount of functional and dedicated tissue-specific studies, e. g., the generation of chromatin interaction maps by Hi-C. Research that addresses this need is substantially more advanced in cancer [20].

## Promises and challenges of whole genome sequencing

Whole genome sequencing (WGS) has the potential to increase the molecular diagnostic rate by addressing several of the limitations outlined above. WGS omits the capture step that is integral to WES, thereby **(i)** reducing the additional time and cost required by the capture and enrichment step and **(ii)** avoiding the capture and hybridization bias, which hampers the reliable detection of CNVs. As the costs of WES and WGS converge, combined with a faster turnaround time for WGS, WGS is set to become the method of choice for generating molecular diagnostic datasets for patients with rare genetic diseases [21].

In contrast to the approximately 50,000 variants found in a WES dataset, the typical WGS dataset yields approximately 3 million small variants, e. g., single-nucleotide variants (SNVs), small indels (< 50 bp), and larger SVs. Large variant frequency population databases, e. g., dbSNP,1http://www.ncbi.nlm.nih.gov/SNP/ gnomAD,2http://gnomad.broadinstitute.org/ 1000 Genomes,3http://www.1000genomes.org/ Decipher,4https://decipher.sanger.ac.uk/ and the 100,000 Genomes Project,5https://www.genomicsengland.co.uk/the-100000-genomes-project/ can help classify the variants into *common* and *potentially disease causing*. Additional databases listing known disease variants and phenotypes, e. g., HGMD6http://www.hgmd.cf.ac.uk/ac/index.php and ClinVar,7https://www.ncbi.nlm.nih.gov/clinvar/ can add additional layers of information [Garda et al. this edition]. This is further supplemented by a myriad of disease-specific databases curated by interest groups. The current classification algorithms for coding region variants are robust due to our understanding of the basic rules of gene transcription and translation. Because we are currently unable to formulate equally overarching and eloquent rules for other aspects of gene regulation, WGS dataset analysis is typically limited to the coding regions and adjacent splice sites. The fact that only 240, or a mere 0.18 %, of the 131,932 variants classified as *pathogenic* or *likely pathogenic* in the ClinVar database as of July 2020 are located outside genes, i. e., in intergenic regions, is a testament to our lack of generalizable rules about how such variants impact the function of non-coding regions.

Our current ability to interpret genomic rearrangements, such as deletions, duplications, inversions, or insertions, collectively called SVs, and variants in the non-coding portions of the genome is severely limited. This means that the majority of genetic variation detected by WGS cannot be consistently and confidently interpreted. Consequently, suspected pathogenic variants in non-coding regions must usually be proven causative through functional studies, tedious and expensive work that typically involves band-shift assays, reporter gene assays, ChIP-seq experiments, and animal models. Because the datasets needed to train classifiers, neuronal networks, and deep learning algorithms are insufficient or missing, we are left without the ability to generate computational models and algorithms for prediction of the effects of genomic rearrangements and variants in the non-coding portion of the genome.

In the articles in this issue the authors illustrate the challenges associated with the analysis of WGS datasets beyond the exonic sequences, including the regulatory genetic machinery. We will provide examples of disease-causing variants from the field of rare genetic diseases of muscle, endocrine, and limb development that help illustrate the various pathogenetic principles and trace the experimental work that was necessary to prove their pathogenicity. The aim of this review article is to provide the readers with a comprehensive understanding of the challenges inherent in interpreting the information that lies beyond the exome, the successes in this field, and the challenges that still lie ahead.

### Mutations located deep in the introns and synonymous mutations

**Figure 1:**
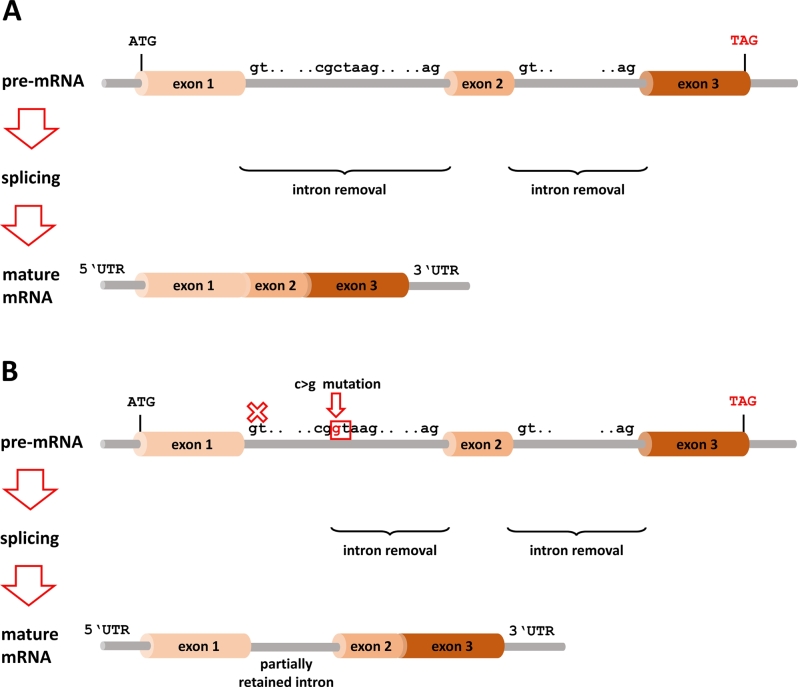
***Splicing defects*****. (A)** Introns are flanked by splice donor sites at the 5′ end and splice acceptor sites at the 3′ end. These sites are marked by special sequence motifs that are highly conserved during evolution. The highest conservation is seen at the “GT..” of the splice donor site and the “..AG” of the splice acceptor site. The other bases around this motif are more variable and determine the strength of a splice site, i. e., the probability that a certain splice site will be used during the splicing process. The spliceosome recognizes these sites in the pre-mRNA and removes the introns between them in order to generate the mature mRNA. **(B)** If a mutation occurs deep inside an intron that generates a strong splice site, e. g., the activation of a cryptic splice site, the splicing mechanism could use this splice site (arrow) instead of the original site (cross). This causes retention of part of the intron, often resulting in a frameshift or truncation of the protein if a stop codon is generated. mRNA, messenger RNA; UTR, untranslated region.

The processing of the transcribed pre-mRNA into mature mRNA is achieved by splicing, i. e., the removal of introns (Figure 1). This complicated process is highly tissue-specific and involves a large number of proteins and small nuclear RNA (snRNA) molecules that form and regulate the megadalton spliceosome complex. Conserved sequence motifs can be found at the 5′ splice donor, the 3′ splice acceptor, and the branch site. The branch site is located within 40–50 bp upstream of the 3′ splice acceptor site and followed by a polypyrimidine tract (reviewed in [22]). Alterations in either of these sequence motifs may lead to mis-splicing of pre-mRNA, subsequent frame-shift mutations or deletions, and, ultimately, compromised protein translation. The effects may be **(i)** ignoring existent splice sites leading to the exclusion of an exon, i. e., exon skipping, or **(ii)** activation of a cryptic splice site inside the intron with incomplete removal of the intron, i. e., intron inclusion. Splicing errors form a significant portion of genetic defects, as shown by the ClinVar database (July 7, 2020) with 8.9 % of “pathogenic” or “likely pathogenic” variants. WES generally covers the splice donor and acceptor sites but is “blind” to deep intronic regions. Exonic variants that do not change the encoded amino acids may still affect splicing. Such alterations can be uncovered only by analyzing mRNA transcripts in parallel with mRNA sequencing. Though this powerful method has recently been developed for a diagnostic setting, it requires access to samples from clearly affected tissues, e. g., liver, muscle, skin, and bone marrow [23].

The cases published by Hamanaka et al. [12] illustrate these aspects nicely. The authors investigated the *NEB* gene in Japanese patients with nemaline myopathy. They found two compound heterozygous pathogenic variants, one deep-intronic variant (c.1569+339A>G, p.Leu524Phefs*9) and one exonic, but synonymous variant (c.24684G>C, p.Ser8228Ser). The intronic variant led to the generation of a new splice acceptor site, thereby including a 67-bp out-of-frame fragment from intron 17 into the mRNA transcript. The synonymous variant was the last exonic variant at the intron 175 splice acceptor site leading to a high proportion of *NEM* transcripts with retained intron 175. Both variants would most likely have been overlooked by WES. Indeed the c.24684G>C variant turned out to be the most frequent pathogenic *NEB* variant in the Japanese population [12].

### Variants in highly repetitive genomic regions

**Figure 2:**
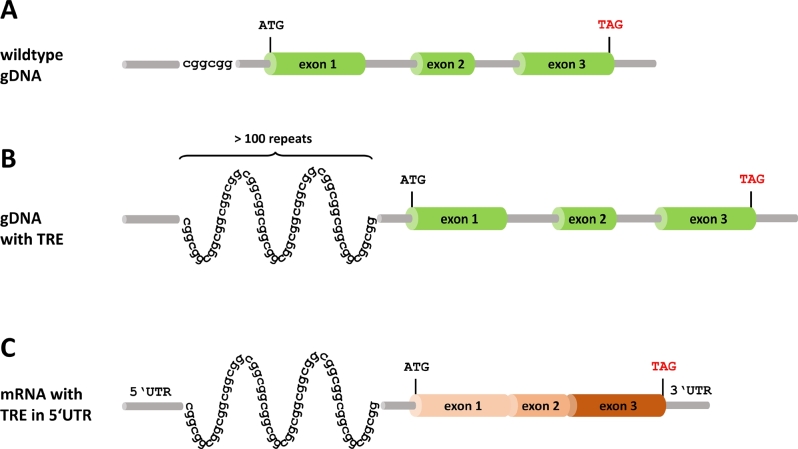
***Triplet repeat extensions*** **(*****TREs*****). (A)** Wild-type gene with non-coding CGG repeats located upstream of the start codon. **(B)** Due to unequal crossing over, such repeat regions can expand in later generations until they comprise hundreds or up to thousands of repeat motifs. **(C)** If this repeat is transcribed into mRNA, the TRE-mRNA may impede the splicing and posttranscriptional processing steps of other mRNAs (mRNA toxicity) and cause disease due to gain of pathologic function of the mRNA. Many of the TRE-related diseases are therefore inherited as dominant traits. gDNA, genomic DNA; mRNA, messenger RNA; TRE, triplet repeat expansion; UTR, untranslated region.

Diseases caused by trinucleotide repeat expansions (TREs) have been known for nearly 30 years, may affect coding and non-coding sequences, and are reviewed in [24]. The non-coding TREs likely impede the processing of other mRNAs (RNA toxicity) (Figure 2). TRE alterations have been considered “unsequenceable” and diagnostic length determination of known, well-described TREs still consists of PCR coupled with fragment length analysis using an electrophoretic method. Long read sequencing [Schwarz et al. this edition] shows promise for the discovery of new TRE-associated disorders [15]. This method can also reveal a TRE as an underlying cause when TREs were not part of the original differential diagnosis. Standard Illumina short read sequence alignments from WES and WGS are not suited for determining the exact number of repeats as they often do not span the entire repeat region. Ishiura et al. [25] addressed this problem with a specific bioinformatic alignment and analysis algorithm of paired-end Illumina reads that uncovered non-coding TREs for the three different disorders: neuronal intranuclear inclusion disease (OMIM #603472), oculopharyngeal myopathy with leukoencephalopathy (OMIM #618637), and oculopharyngodistal myopathy (OMIM #164310). As all these disorders share the same phenotype and repeat motif, the authors hypothesized that the disease phenotype does not depend on the affected gene, but on the repeat motif *per se* [25].

### Variants impacting promoters and enhancers

The disease-causing variants that lead to developmental defects of endocrine glands illustrate the promises and challenges of WGS. Congenital endocrine diseases, such as congenital hypothyroidism (CH) and congenital diabetes mellitus (CDM), are well-suited disease models because they usually only affect one cell type that secretes a specific hormone. This stands in contrast to genetic defects affecting more complex organs, such as the liver or brain. The gene networks involved in organ development and hormone synthesis are therefore less complex and most of the disease-causing variants known today were identified 15 years ago, e. g., variants in the approximately 20 genes for CH [26] and in the approximately 25 genes for CDM [27]. The known variants explain 80 % of the CDM cases, but only 5 % of the CH cases that are due to thyroid dysgenesis (CHTD), leaving a large proportion of patients without a molecular diagnosis. It is likely that mutations in these patients are located outside of the coding regions and are therefore missed by WES. Only a very limited number of cases with CHTD have been diagnosed at the time of writing.

Due to our comprehensive understanding of the physiology and pathways of the endocrine system, this disease group is useful when demonstrating the added value of WGS in identifying non-coding disease-causing variants in patients with suspected rare genetic diseases. We will use examples from non-coding disease-causing alterations in the *PAX8* and *NKX2-1* genes causing CHTD, and in *PTF1A* causing CDM, to illustrate the usefulness of this approach.

The TFs PAX8 and NKX2-1 are key regulators of early thyroid development. Disease-causing variants in their coding regions are well-documented causes of CHTD [28], [29]. A lack of understanding of how expression of these TFs is regulated during early thyroid development has limited our ability to successfully identify disease-causing variants in the non-coding regulatory regions of these genes. Perone et al. [30] identified an SNV in the *PAX8* promoter that is located 1 kb upstream of the transcription start site in a patient with CHTD. As shown by functional luciferase and gel mobility shift assays, this variant interferes with *PAX8* transcription [30]. Though a few, small deletions that may impact *NKX2-1* transcription have been identified, the functional relevance of these deletions has not been proven, because the enhancer sequences that drive *NKX2-1* expression during thyroid development are still unknown [31]. This illustrates the requirement for organ- and developmental stage-specific knowledge of regulatory networks and elements before such SVs can be reliably interpreted.

**Figure 3:**
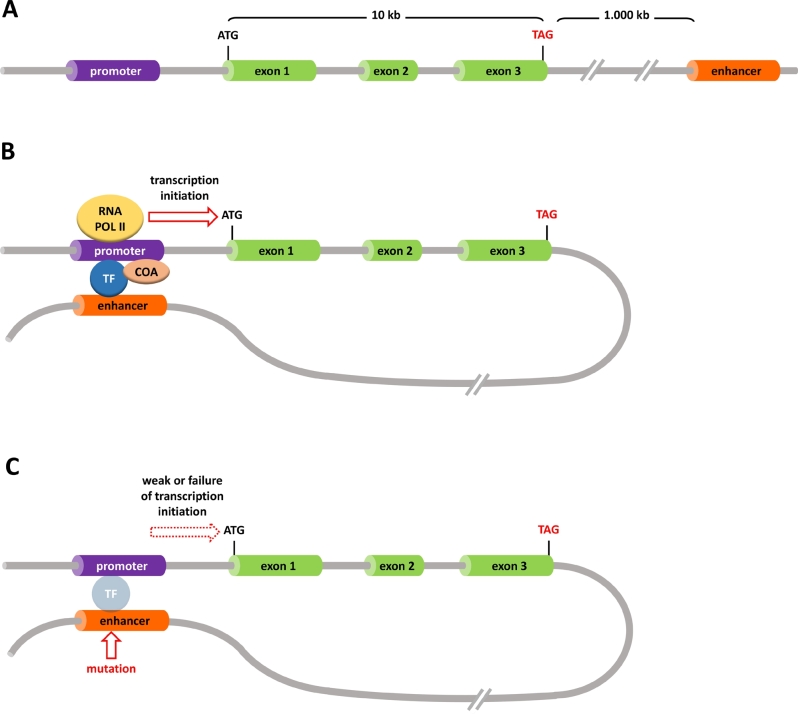
***Schematic depiction of a gene with its promoter and an enhancer*****. (A)** Linear arrangement of the promoter that is always located at the 5′ position of a gene consisting of three exons. Enhancer elements may be found either up- or downstream of the gene and can be separated by hundreds or even thousands of kb of DNA from the gene they act on. **(B)** Enhancer and promoter come into proximity by looping. This facilitates the binding of TFs and coactivators (COA). Ultimately, RNA polymerase II (RNA POL II) is recruited to this transcription initiation complex and the specific gene will be transcribed into pre-mRNA. **(C)** In the case of an enhancer mutation, e. g., missense mutation or deletion, the TFs cannot bind properly, the transcription initiation complex is not formed, and specific mRNA will be synthesized in insufficient quantities or not at all.

These uncertainties in the pathogenesis of CHTD stand in stark contrast to an example of CDM reported for the *PF1A* gene locus. Sellick et al. [32] reported autosomal recessive coding variants in *PF1A* as the cause of CDM with cerebellar agenesis and severe CNS symptoms [32]. Following a systematic approach for identifying the genetic cause of isolated CDM secondary to pancreatic agenesis without CNS dysfunction in multiple consanguineous families, Weedon et al. [33] discovered a specific enhancer for PTF1A expression that is active exclusively during early embryonic pancreatic development. The combination of autozygosity mapping and WGS identified a homozygous variant that co-segregated with CDM and was located 25 kb downstream of the last *PTF1A* coding exon. To complement these findings, the authors performed a genome-wide search for active enhancer sequences in endodermal pancreatic progenitor cells, identified more than 6,000 active enhancer sequences, and determined that the homozygous variant of interest was located within an early pancreatic development-specific active enhancer. Further functional studies demonstrated the loss of *PTF1A* transcription in the presence of the co-segregating variant [33]. Subsequently, the authors found three additional loss-of-function variants and one small deletion in the same *PTF1A* downstream enhancer, lending further proof to their initial findings [33] (Figure 3). The *PTF1A* enhancer variants discussed above illustrate how the phenotypic spectrum displayed by patients with disease-causing variants in non-coding regulatory regions can differ from patients where the cause is located in the coding region.

At the time of discovery, data on active chromatin regions from the ENCODE project did not point towards an enhancer function of the sequence where the variant was located. Only the tissue- and developmental time point-specific enhancer search in endodermal pancreatic precursor cells uncovered the relevant active *PTF1A* enhancer. This demonstrates the painstaking groundwork necessary to interpret non-coding variants. Such basic studies have to be performed in specific tissues as well as during different stages of organ development. In conclusion, the authors would have been unable to pinpoint the disease-causing *PTF1A* variant had they not investigated the informative consanguineous families with the same homozygous (founder) disease-causing variant by the classical method of positional cloning. Disease-causing variants in the *PTF1A* enhancer are now the most frequent genetic cause of isolated pancreatic agenesis [34]. This example clearly illustrates how loss-of-function enhancer variants can explain the disease phenotype in a large subset of “WES-negative” patients, highlighting the added value that WGS can bring to these patients.

### Detection and interpretation of structural variants

The term structural variation (SV) comprises balanced rearrangements, also referred to as copy number variations (CNVs), as well as unbalanced genomic rearrangements including inversions, insertions, and translocations. While larger CNVs can be detected efficiently by array-CGH, balanced rearrangements were classically only detected by cytogenetics. In principle, WGS offers the opportunity to detect all classes of SVs in a genome-wide manner. One important limitation is the impossibility to unambiguously map reads in highly repetitive regions of the genome. As genomic breakpoints frequently map to these regions, this limitation poses a significant problem. The detection of SVs by WGS in a diagnostic setting therefore poses a challenge that cannot be fully overcome with short read Illumina technology.

Long single-molecule sequencing technologies produce reads of 10–50 kb that are more likely to span repetitive regions and can therefore be used to reliably map breakpoints. Further methods to resolve complex SVs and rearrangements comprise Hi-C and optical mapping; see Schwarz et al. (this edition). Careful evaluation of the breakpoints with these technologies often reveals additional changes, such as smaller deletions, inversions, or insertions [34], that are exceedingly difficult to detect with standard WGS short read sequencing. Information about the precise breakpoints and the full characteristics of each SV is needed in order to interpret its effect.

**Figure 4:**
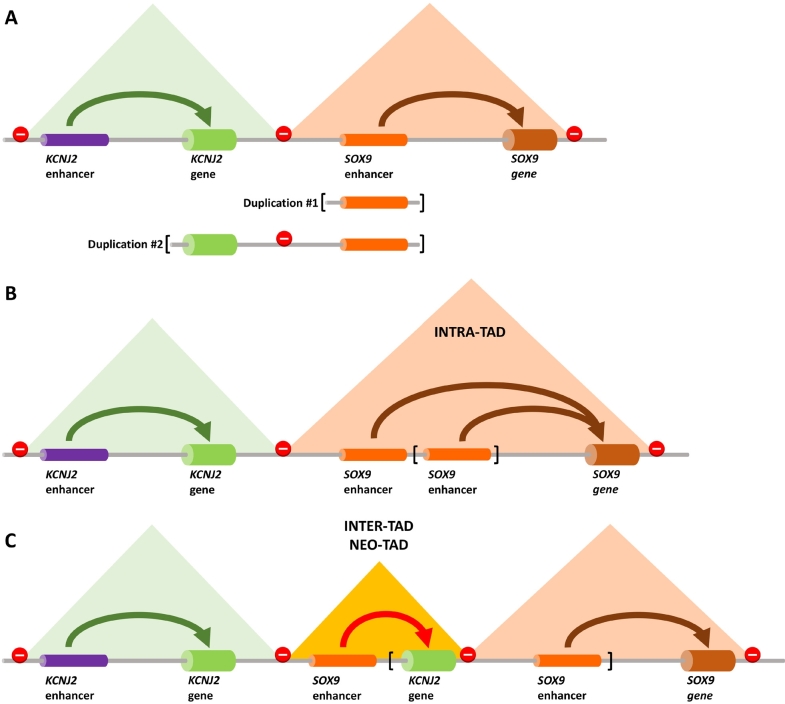
*Two neighboring TADs of the KCNJ2 and SOX9 genes with their respective enhancers*. **(A)** The arrows depict the interaction between the gene and its enhancer. No interactions are seen between enhancers and genes from neighboring TADs. The stop signs depict the TAD boundaries, mostly characterized by binding of the CTCF TF. The pyramids depict the TAD interactions as revealed by Hi-C [Guo *et al*. this issue]. **(B)** In the case of an intra-TAD duplication (Duplication #1) in the *SOX9* TAD, enhancer elements are increased, leading to stronger *SOX9* activation and subsequent sex reversal. **(C)** If the duplication contains a TAD boundary (Duplication #2), a neo-TAD will be formed. In this case the *KCNJ2* gene comes under the influence of the *SOX9* enhancer, leading to limb malformation (Cooks syndrome).

Detected SVs need to be evaluated with respect to their potential pathogenicity. This involves several steps as summarized in Spielmann et al. [19]. Several large initiatives, such as the 100,000 Genomes Project and the Deciphering Developmental Disorders (DDD) study, have evaluated the number of *de novo* SVs in a variety of patient cohorts. Such CNV morbidity maps have proven extremely useful for the clinical interpretation of CNVs [1]. One of the most comprehensive SV databases, the Database of Genomic Variants (DGV),8http://dgv.tcag.ca/dgv/app/home provides a catalogue of SVs found in the genomes of control individuals from diverse populations [34]. In the case of CNVs, the pathogenic effect of gene dosage has to be considered. Many genes are dosage sensitive either to haploinsufficiency or to increased dosage due to duplications. Whether or not this is the case for any of the genes included in the CNV can be determined with information from current databases, such as the DDD database.9https://decipher.sanger.ac.uk/ddd/overview The gene dosage approach has been successful when determining the etiology of several recurrent microdeletion syndromes, such as the 17q21.31 deletion syndrome caused by haploinsufficiency of *KANSL1* [35] or the 17p11.2 microdeletion associated with Smith–Magenis syndrome (OMIM #182290) resulting from haploinsufficiency of *RAI1* [36]. In other cases, deletions and reciprocal duplications can result in different phenotypes as described at the chromosomal region 17p12, where duplications result in Charcot–Marie–Tooth disease type 1A (OMIM #118220) [37], whereas deletions cause hereditary neuropathy with liability to pressure palsies (OMIM #162500) [38].

If the CNV does not contain a dosage sensitive gene that can explain the phenotype, or if the CNV covers only non-coding sequences, other possibilities may be considered. In a series of studies we showed that CNVs and balanced SVs can result in gene mis-expression by changing the chromatin landscape [19]. In these cases, the structural rearrangements lead to a rewiring of enhancer–promoter contacts and subsequent gene mis-expression.

### Variants affecting chromatin configuration and enhancer–promotor crosstalk

Gene expression levels are regulated on multiple levels, most prominently at the promoter and enhancer levels. While promoters are always located at the 5′ end of a gene, where they function in a direction dependent manner, enhancers can be located far removed from their target gene (> 1 Mb) and are thought to function in a largely position independent manner. Enhancers are important for the fine-tuning of expression, in particular in situations that demand a high degree of precision, e. g., during development. Enhancers interact with their cognate promoters *via* looping, i. e., the regulatory elements come into close proximity with each other even if they are distant on the linear genome. Looping involves highly regulated chromatin folding and it plays an important role in gene regulation. Which regions of chromatin interact with each other is determined by chromatin domains, defined as regions of high interactions that are separated by regions with low interaction. These domains, called topologically associating domains (TADs), have been identified genome-wide by chromatin conformation capture technologies, in particular Hi-C. These technologies are reviewed by Guo et al. (this edition).

SVs that disrupt TAD integrity can result in disease. The regulatory activity contained in a TAD is separated from its neighbor by a boundary region. The regions typically feature multiple binding sites for the looping-promoting TF CTCF, thereby insulating neighboring activities. Deletion of such a boundary can lead to a fusion of two TADs. The subsequent aberrant enhancer–promoter contacts can, in turn, lead to gene mis-expression. This mechanism was originally described at the *EPHA4* locus, where the *EPHA4* enhancer activates the neighboring *PAX3* gene after deletion of the boundary [39].

Duplications containing a boundary can, on the other hand, result in the formation of novel chromatin domains (Figure 4). This has been described at the *SOX9* locus, where large duplications upstream of *SOX9* result in activation of the neighboring gene *KCNJ2* and consecutive limb malformation. In contrast, duplications that do not contain the boundary result in sex reversal due to aberrant activation of *SOX9* (Figure 4) [11]. The difference between both abnormalities lies in the structure of the chromatin domains: in the case of limb malformation (Cooks syndrome, OMIM #106995), a new chromatin domain is formed that insulates the duplication from *SOX9* (Figure 4), whereas in sex reversal (OMIM #278850) the duplication resides within the *SOX9* TAD and therefore affects its expression. The mechanisms of gene regulation alteration following disruption of chromatin domains have been reviewed in [40].

## Conclusion & outlook

Our improved ability to consistently and reliably interpret non-coding variants depends on the availability of specific data and our ability to understand said data in the context of rare genetic diseases. In order to have a complete picture of the genome for each individual patient, i. e., a picture that includes repetitive sequences and SVs, we will need access to affordable and reliable long read sequencing. In order to understand the impact of non-coding variants on gene expression, we need to have data on all regulatory elements, e. g., enhancers, insulators, TFBSs connected to the loci of interest, and the chromatin state of these regulatory elements in the primarily affected tissues, cell types, and different developmental stages, as well as RNA sequencing data. This information will need to be interpreted in the context of allelic distribution, meaning that we will need reliable population data. Finally, we will need user-friendly software tools that integrate all of the data described above. Many researchers, ourselves included, are working to contribute to all of the above, with the ultimate aim to end the diagnostic odyssey for all patients with rare genetic diseases.
